# Sexual distress among men with cancer – a cross-sectional study

**DOI:** 10.2340/1651-226X.2025.42525

**Published:** 2025-02-09

**Authors:** Jonas Nahavandipour, Christoffer Johansen, Annamaria Giraldi, Bolette Skjødt Rafn, Annika von Heymann

**Affiliations:** a Danish Cancer Society National Cancer Survivorship and Late Effects Research Center (CASTLE), Department of Oncology, Rigshospitalet, Copenhagen University Hospital, Copenhagen, Denmark; b Sexological Clinic, Mental Health Center Copenhagen, Mental Health Services Capital Region, Copenhagen, Denmark; c Department of Clinical Medicine, University of Copenhagen, Copenhagen, Denmark

**Keywords:** Sexual distress, sexual function, neoplasms, health related quality of life, late effects

## Abstract

**Background and purpose:**

Many men with cancer experience that changes created by cancer and its treatment may impair sexual function. However, many studies investigating sexual impairments fail to consider whether such impairments are perceived as distressing, i.e. create sexual distress. We investigated the prevalence of sexual distress, overlap with sexual impairment, and sociodemographic and clinical characteristics and other symptoms associated with sexual distress in a heterogeneous male cancer population.

**Patients and methods:**

Across cancer diagnoses, 2792 men in treatment or follow up at the Department of Oncology, Rigshospitalet, were invited. The Sexual Complaint Screener (SCS) assessed sexual impairments and sexual distress. Regression analyses estimated the association of sexual distress with sociodemographic and tumor-related factors, other symptoms (pain, depression, fatigue, insomnia, fear of recurrence), and health-related quality of life. The number of patients who received help for or were interested in a consultation for sexual problems was calculated.

**Results:**

Six hundred and ninety-six patients, most frequently diagnosed with testicular (26%) or multiple (16%) cancers, completed the SCS. Forty-one per cent experienced sexual distress, 60% sexual impairment, and 34% overlapping sexual distress and impairment. Sexual distress was significantly associated with clinically relevant insomnia (OR:2.15; 95% CI:1.5–3.1) and pain (OR:1.90; 95% CI:1.3–2.9). Two thirds of all patients wished for help, but only one third of these were receiving help.

**Interpretation:**

Sexual distress was widespread in men across different cancer diagnoses and sometimes presented without impairment, demonstrating that assessment of sexual problems must include the personal experience of distress and extend to men across cancer diagnoses.

## Introduction

**Figure 1:**
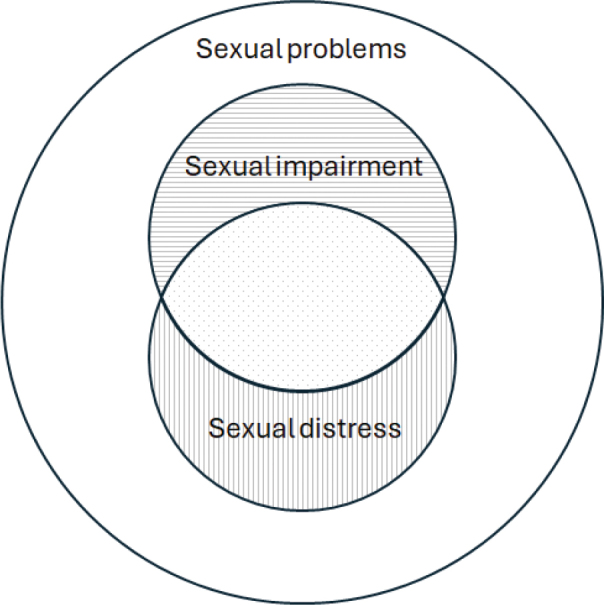
Illustration of sexual problems and the overlap between sexual impairment and sexual distress. Dotted area corresponds to reports of sexual impairments, combined with sexual distress.

Cancer and its treatment can create many complex side and late effects which can lead to impaired sexual function across different areas, such as reduced desire, difficulty achieving erection, ejaculation/orgasm problems or pain related to intercourse [1, 2]. Sexual impairment significantly impacts health-related quality of life (HRQoL) [3–5], and may be more than five times as frequent among male cancer survivors than among healthy individuals [6]. When sexual impairments are perceived by the individual to be distressing, this is termed sexual distress (or bother) [7], and impairment can be present without sexual distress (Figure 1) as demonstrated in populations with prostate cancer (e.g. Bravi et al. [8]). The presence of sexual impairment alone may thus not be a clear indicator of need for support, and some studies have shown that sexual distress may be more strongly associated with HRQoL than impairment [3, 4]. Even though sexual impairment has been documented for men with many different cancer diagnoses [9–11], sexual distress has been very sparingly assessed in men with cancer diagnoses other than prostate cancer [5, 12–15].

We therefore investigated the prevalence of sexual distress and the degree to which it was reported with or without impairment among men with all types of cancer seen in a large oncology department. To characterize patients reporting sexual distress, we investigated the association of demographic and clinical characteristics as well as other symptoms that are potential side or late effects of cancer (depression, fatigue, fear of recurrence, insomnia, and pain) with reporting sexual distress, and to clarify a potential effect of sexual distress on HRQoL, we investigated their association. Finally, we calculated the number of patients who reported that they received help for sexual problems and the number who were interested in a consultation for sexual problems, and their overlap with numbers reporting sexual distress.

## Methods

This study utilizes data from a large cross-sectional questionnaire study conducted from February until April 2021 at the Department of Oncology, Copenhagen University Hospital Rigshospitalet, Denmark, approved by the local research legal department (reference 23039798). All patients across diagnoses in active cancer treatment or in a follow-up program, at any time since diagnosis, were invited to complete an online questionnaire through the national secure digital mail ‘e-Boks’. Following informed consent, data was collected and processed according to applicable data protection regulations. In the present analyses, we included only men aged ≥18 and <100 years with access to digital secure mailbox, and complete response to questions on sexual problems.

Participants self-reported sociodemographic and lifestyle characteristics (age, sex, education, cohabitation, employment, alcohol consumption, and tobacco smoking) and clinical characteristics (cancer diagnosis, time since first diagnosis, and treatment received [surgery, radiation, chemo-, immune-, and/or hormone therapy]). Self-reported cancer diagnoses were cross-checked with medical records. Patients with diagnoses reported by fewer than 20 respondents were grouped, as were those reporting multiple cancers.

## Instruments

The male version of the Sexual Complaint Screener (SCS-M) is a 10-item patient-reported questionnaire developed by the International Society for Sexual Medicine to screen all major areas of sexual function and distress within the past 6 months [16]. It consists of seven items evaluating sexual impairment, one item evaluating satisfaction with sexual life and one item assessing interest in consultation for sexual problems. Items included here evaluated desire, ability to maintain an erection, need for increased stimulation, experiencing early ejaculation, the lack of orgasm or ejaculation, and pain during or after intercourse. The frequency of experiencing each sexual impairment is reported on a five-point Likert scale (‘Never/almost never’; ‘Rarely’; ‘Sometimes’; ‘Often’; ‘Almost all the time/Always’), unless ‘No sexual activity’ is selected. Each question about sexual impairment is accompanied by a question assessing the severity of distress experienced due to this impairment with responses on a five-point Likert scale (‘Not at all a problem’; ‘A very small problem’; ‘Some problem’; ‘A considerable problem’; ‘A very great problem’). The SCS-M does not have a predefined cut-off to indicate clinical relevance. Therefore, to determine the presence of impairment, regardless of its magnitude, we defined sexual impairment to be present, when it was reported with responses of ‘Sometimes’ or more frequently, and sexual distress as present at responses of ‘Some problem’ or more. Further, we included the item assessing interest in a consultation about sexual problems (Response options: ‘No’, ‘Not now’, and ‘Yes’). The SCS-M was developed in Danish and Swedish and translated to English. It has been preliminarily validated [17], used in studies of male populations with e.g. kidney diseases [18], but not yet validated in Danish. Finally, we used a self-constructed item asking if participants were currently receiving help for a list of issues (yes/no for e.g. depression or fatigue), among these sexual problems.

The remaining questionnaires assessed HRQoL and other symptoms that are potential side or late effects of cancer and its treatment. All questionnaires have previously been used or validated among Danish cancer patients (see Supplement for references).

HRQoL within the last week was assessed using the overall, physical, and emotional subscales of the European Organization for Research and Treatment of Cancer Quality of Life Questionnaire Core 30 (EORTC QLQ-C30). Each sub-score is reported as the transformed score between 0 and 100 with higher scores reflecting better function [19]. To determine clinical relevance, differences of 5–10 were considered small, 10–20 moderate, and >20 large, as suggested by Osoba et al. [20].

Symptoms of depression within the last 2 weeks were measured using the 12-item Major Depression Inventory (MDI) returning scores between 0 and 50. A score of ≥21, indicative of minor depression or worse, was used as a cut-off value for clinically relevant depression [21].

Symptoms of fatigue experienced ‘lately’ were measured with the 20-item Multidimensional Fatigue Inventory-20 (MFI-20) [22]. The general fatigue score, ranging from 4 to 20 with higher scores indicating greater fatigue, was dichotomized at the 75th percentile as a cut-off to indicate clinically relevant fatigue [23].

Fear of recurrence within the last month was measured with the nine item Fear of Cancer Recurrence Inventory (FCRI)-short form. It returns a total score between 0 and 36, with higher scores reflecting worse functioning [24], and a cut-off of ≥22 was used to determine clinically relevant symptoms [25].

The seven item Insomnia Severity Index (ISI) questionnaire assessed symptoms of insomnia with the last month. Possible scores were between 0 and 28, with higher scores indicating greater insomnia [26]. A score of ≥ 8 was used as cut-off for clinically relevant symptoms [27].

Pain during the last 24 hours was assessed using the pain interference (PITS) subscale of the Brief Pain Inventory (BPI) [28]. The PITS score is calculated as a mean of seven items scored on a scale from 0 to 10 measuring interferences with daily activities caused by pain. A cut-off of ≥ 2, indicative of moderate to severe pain, was used [29].

### Analysis

Differences in demographic and clinical characteristics between those who completed the SCS-M and those who did not were investigated using logistic regression. Age was divided into quartiles, because of non-linear relationship to the log odds of responding.

To investigate the overall prevalence of sexual distress and its co-occurrence with impairment, the number of participants was calculated who reported sexual distress on one or more SCS-M items (regardless of reporting impairment or not), sexual impairment on one or more items (regardless of reporting distress or not), and distress combined with impairment on one or more items. For each SCS-M item, we calculated the number of participants reporting all possible combinations of distress (yes/no) and impairment (yes/no). Post-hoc analyses were performed for patients in and not in active treatment. Further, patients were grouped by the number of areas in which they experienced distress, and the percentage of participants with interest in consultation for sexual problems was calculated for each group.

**Figure 2:**
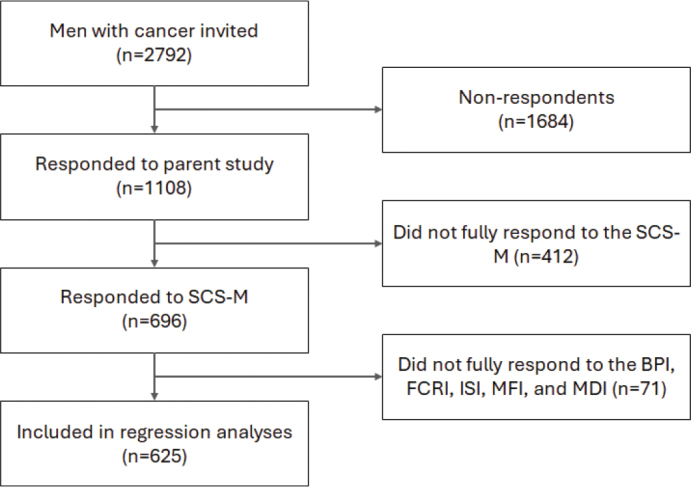
Flowchart for inclusion and exclusion of respondents. BPI: Brief Pain Inventory; FCRI: Fear of Cancer Recurrence Inventory; ISI: Insomnia Severity Index; MDI: Major Depression Inventory; MFI: Multidimensional Fatigue Inventory; SCS-M: Sexual Complaints Screener-Male.

To investigate the characteristics associated with experiencing sexual distress, a multivariable logistic regression was performed with distress in any area as the dependent variable, while independent variables were other symptoms, sociodemographic, and clinical characteristics, all mutually adjusted. In post-hoc analyses, we ran this logistic regression separately for subgroups of participants currently receiving and not receiving treatment. To investigate the correlation between HRQoL and sexual distress in any area, linear regressions were performed. For each domain of HRQoL (overall, physical, and emotional), three models were run: (1) crude; (2) adjusted for sociodemographic and clinical characteristics by adding age, cohabitation, employment, cancer site and time since diagnosis; (3) adjusted for sociodemographic, clinical characteristics, and other symptoms, by further adding dichotomized scores for clinically relevant pain, fatigue, depression, fear of recurrence, and insomnia. Because of indication of non-linear relationship, we performed sensitivity analysis with generalized additive models (GAM), with clinically irrelevant differences between estimates in linear models and GAMs (data not shown), substantiating the robustness of the linear analyses. Regression analyses were performed on participants with complete responses to the SCS-M, the EORTC QLQ-C30, MFI-20, FCRI, ISI, and BPI (Figure 2). Outliers and respondents with improbable scores were removed. Analyses were conducted using R version 4.1.0. [30], with the *geepack* package for linear and logistic analysis, *mgcv* for GAM-models, and *ggplot2* for graphs.

## Results

Of 2792 male patients invited to the parent study, 1108 responded (40%) and, of these, 696 (63%) completed the optional SCS-M (Figure 2). Of respondents to the parent study, older patients and patients with esophageal or stomach cancers, brain cancers, or multiple cancers were less likely to respond to the SCS-M, while patients receiving help for sexual problems were more likely to respond (Supplement, Table S1). SCS-M respondents’ mean age was 60 years and 76% were cohabitating (*n* = 530, Table 1). The most prevalent cancer type was testicular cancer (*n* = 183, 26%), followed by patients with multiple cancers (*n* = 108, 16%), or other cancers (*n* = 75, 11%), with a mean time since first diagnosis of 0.9 years (Table 1). The majority (78%) of patients were not currently receiving antineoplastic treatment.

**Table 1:** Characteristics of men with cancer in treatment or follow up at the Department of Oncology, Rigshospitalet who responded to the Sexual Complaint Screener (SCS-M) (*n* = 696).

**Age (years)**	
Mean (SD)	60 (15)
Min – Max	19–89
**Living alone (*n*, %)**	
Yes	166 (24%)
**Level of education (*n*, %)**	
Primary school	65 (9%)
High school	33 (5%)
Continued education (1–2 years)	84 (12%)
Continued education (3–4 years)	270 (39%)
Higher education (> 4 years)	204 (29%)
Others	40 (6%)
**Employment (*n*, %)**	
Full-time employment	243 (35%)
Part-time employment	33 (5%)
Self-employed	53 (8%)
Unemployed	25 (4%)
Retired	258 (37%)
On sick leave	50 (7%)
Others*	32 (5%)
Missing	2 (0.3%)
**Primary cancer site (*n*, %)**	
Testicular	183 (26%)
Head and neck	69 (10%)
Esophageal/stomach	57 (8%)
Lung	51 (7%)
Prostate	51 (7%)
Brain	41 (6%)
Colorectal	41 (6%)
Bladder	20 (3%)
Multiple	108 (16%)
Others	75 (11%)
**Cancer treatment (*n*, %)**	
Surgery	
Yes	486 (70%)
Chemotherapy	
Received	467 (67%)
Not received	223 (32%)
Do not know	5 (0.7%)
Missing	1 (0.1%)
Radiotherapy	
Received	307 (44%)
Not received	377 (54%)
Do not know	7 (1%)
Missing	5 (0.7%)
Hormone therapy	
Received	72 (10%)
Not received	587 (84%)
Do not know	29 (4%)
Missing	8 (1%)
Immunotherapy	
Received	592 (85%)
Not received	42 (6%)
Do not know	57 (8%)
Missing	5 (0.7%)
Currently receiving treatment**	
Yes	151 (22%)
**Time since first cancer diagnosis (years)**	
Mean (SD)	0.9 (0.3)
Missing (*n*, %)	47 (7%)
**Reported receiving help for sexual dysfunction (*n*, %)**	
Yes	65 (9%)
No	643 (90%)
Missing	2 (0.3%)
**Presence of other clinically significant potential side or late effects (*n*, %)**	
Depression	91 (13%)
Missing	1 (< 1%)
Fatigue	142 (20.4%)
Missing	9 (1.3%)
Pain	143 (21%)
Missing	5 (< 1%)
Insomnia	336 (48%)
Missing	0 (0%)
Fear of recurrence	162 (23%)
Missing	15 (2.2%)

* Other occupations: Students and staying-at-home.

** Chemotherapy, immunotherapy and radiotherapy.

**Figure 3:**
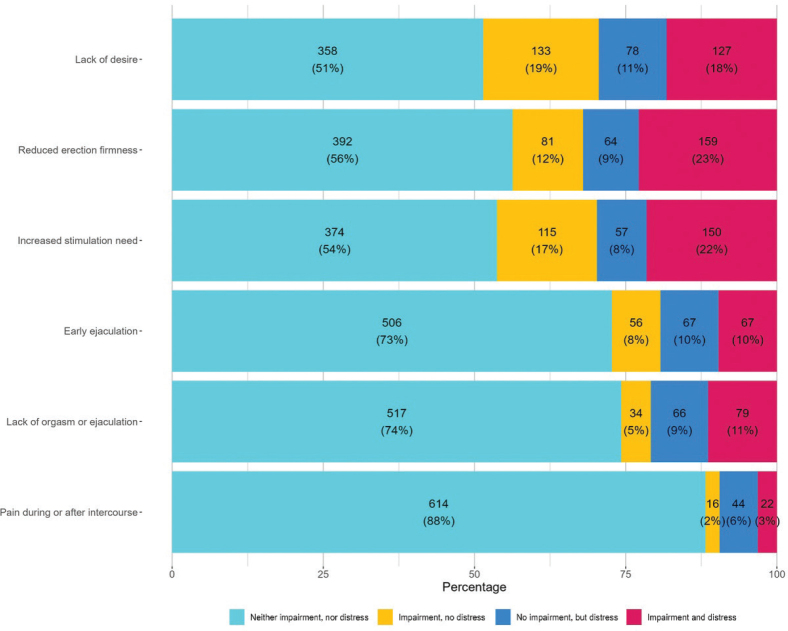
Distribution of sexual distress and impairment among 696 men with cancer at the Department of Oncology, Rigshospitalet.

Overall, 288 (41%) of the 696 respondents reported sexual distress on one or more items (the vertically lined and dotted areas of Figure 1), 420 (60%) reported sexual impairment on at least one item (horizontally lined and dotted areas), and 234 (34%) reported both sexual distress *and* sexual impairment on one or more items (dotted area alone). When inspecting each item individually, a sexual impairment that was *not* experienced as distressing was reported by between 2% (pain during or after intercourse) and 19% (lack of desire) (Figure 3). Between 6% (pain during or after intercourse) and 11% (lack of desire) reported sexual distress without reporting a sexual impairment, and of these (Figure 3), the majority responded that they were not sexually active (60–82% per item). The combination of sexual impairment *and* distress was reported by between 3% (pain during or after intercourse) and 23% (reduced erection firmness) per item (Figure 3, Supplement Table S, Figure S1). A similar pattern was observed in post-hoc analyses among participants in treatment and not in treatment (See Supplement Figure S2, Figure S3, and Table S3)

When investigating factors associated with reporting sexual distress on at least one item, we found that only clinically relevant insomnia (OR = 2.2; 95% CI:1.48–3.11) and pain (OR = 1.9; 95% CI:1.25–2.91) were significantly associated with experiencing sexual distress (Table 2). This remained unchanged in both post-hoc analyses, while only higher education (1–2 years) among patients in treatment emerged as a new associated factor (see Supplements Table S4 and S5). In fully adjusted analyses, sexual distress was significantly associated with lower overall and emotional HRQoL, but not physical HRQoL. Respondents experiencing distress reported –3.2 (95% CI: –5.6 to –0.8) lower overall HRQoL scores and –3.8 (95% CI: –6.3 to –1.3) lower emotional HRQoL score (Table 3).

**Table 2:** Odds ratios for sexual distress among men with cancer in follow up or treatment at the Department of Oncology, Rigshospitalet (*n* = 625).

Patient characteristics and symptoms	OR* (95% CI**)	*P*
**Age (years)**	1.0 (0.98–1.01)	0.39
**Living alone**		
No	Ref	
Yes	0.9 (0.60–1.37)	0.64
**Level of education**		
Higher education (> 4 years)	Ref	
Higher education (3–4 years)	1.0 (0.65–1.47)	0.90
Higher education (1–2 years)	0.7 (0.39–1.38)	0.34
High school	0.7 (0.30–1.78)	0.49
Primary school	1.1 (0.56–2.02)	0.84
Others	1.5 (0.67–3.38)	0.33
**Primary cancer site**		
Testicular	0.7 (0.29–1.59)	0.38
Head and neck	0.5 (0.20–1.18)	0.11
Esophageal/stomach	0.9 (0.38–2.18)	0.84
Lung	0.5 (0.19–1.13)	0.15
Prostate	Ref	
Brain	0.8 (0.31–2.02)	0.63
Colorectal	0.7 (0.29–1.90)	0.54
Bladder	1.3 (0.48–3.74)	0.58
Multiple	1.0 (0.46–2.24)	0.97
Others	0.5 (0.23–1.25)	0.15
**Time since first cancer diagnosis (years)**	1.0 (0.99–1.07)	0.21
**Pain**		
Present	1.9 (1.25–2.91)	< 0.01
**Fatigue**		
Present	0.7 (0.42–1.10)	0.11
**Depression**		
Present	1.5 (0.87–2.71)	0.18
**Fear of recurrence**		
Present	1.3 (0.84–1.98)	0.25
**Insomnia**		
Present	2.2 (1.48–3.11)	< 0.01

* Mutually adjusted,

** Confidence intervals.

**Table 3:** Association between health-related quality of life (EORTC-QLQ-C30) and sexual distress among men with cancer in follow up or treatment at the Department of Oncology, Rigshospitalet (*n* = 625).

Health related quality of life (HRQoL) domain	Coefficients	*P*
**Overall (**HRQoL)	Beta (95% CI)	*P*-value
1 Crude Analysis	–8.5 (–11.7 to –5.3)	< 0.01
2 Adjusted for sociodemographic* and clinical characteristics**	–7.7 (–10.7 to –4.6)	< 0.01
3 Adjusted for sociodemographic, clinical characteristics and other symptoms***	–3.2 (–5.6 to –0.8)	0.01
**Emotional (**HRQoL**)**		
1 Crude Analysis	–8.7 (–12.0 to –5.5)	< 0.01
2 Adjusted for sociodemographic and clinical characteristics	–8.1 (–11.2 to –5.0)	< 0.01
3 Adjusted for sociodemographic, clinical characteristics and other symptoms	–3.8 (–6.3 to –1.3)	< 0.01
**Physical (**HRQoL)		
1 Crude Analysis	–1.8 (–4.6 to 1.0)	0.20
2 Adjusted for sociodemographic and clinical characteristics	–1.3 (–3.8 to 1.2)	0.32
3 Adjusted for sociodemographic, clinical characteristics and other symptoms	1.7 (–0.6 to 3.9)	0.16

* Sociodemographic characteristics: cohabitation, level of education and age.

** Clinical characteristics: cancer site and time since diagnosis.

*** Symptoms: Pain, fatigue, depression, fear of recurrence and insomnia.

EORTC-QLQ-C30: European Organization for Research and Treatment of Cancer Quality of Life Questionnaire Core 30.

Nine percent (*n* = 65) of the SCS-M-respondents reported that they received help for sexual problems, 28% (*n* = 192) were interested in receiving a consultation about sexual problems at the time of response, and 34% (*n* = 236) at a later point. Interest in consultation for sexual problems was more frequently endorsed by patients experiencing distress in more areas (Supplement, Figure S4).

## Discussion

To the best of our knowledge, this is the first study to investigate the prevalence of sexual distress, associated sociodemographic and clinical factors, and the potential impact of sexual distress on HRQoL in a heterogeneous population of men with cancer. In our sample of men with diverse cancers, 60% reported a sexual impairment, 41% experienced sexual distress, and 34% reported the combination of both impairment and distress in at least one area. Sexual distress was significantly associated with clinical levels of pain and insomnia. More than two thirds of participants were interested in a consultation for sexual problems.

We found that sexual distress was prevalent, reported by 41% in at least one area. Sexual distress is most widely documented among men with prostate cancer, and among the largest studies is an Australian study that reported the prevalence of sexual distress between 29 and 43% 12 months after treatment, greatest after radical prostatectomy [31]. Meanwhile, a systematic review of 81 studies found prevalence rates up to 88% [1]. Among other male cancer populations, estimates of sexual distress are scarce. A Japanese study reported the prevalence among patients who had undergone bladder cancer surgery (74% men) to be between 25 and 74% for different sexual areas, highest for ability to have intercourse [12]. A Danish study, conceptualizing sexual distress broadly as negative feelings about sexual problems, e.g. including guilt or feelings of inferiority, found sexual distress in 42% of men with hematological cancers [13]. The prevalence found in the present heterogeneous population falls within the range previously reported for individual cancers, underscoring that sexual distress may be widespread in men across diagnoses. Indeed, cancer type was not found to be associated with sexual distress. Some studies have calculated changes in sexual distress over time, rather than prevalences, most reporting increased distress after treatment [32] and improvement over time [33] in men with prostate cancer, while some report distress scores to be stable [4, 5], including one study in colorectal cancer [5]. Our findings agree with part of this evidence, as time since diagnosis was not found to be associated with distress, although this finding should be interpreted with caution due to the cross-sectional design.

We found that men who reported clinically relevant pain and insomnia were more likely to report sexual distress, which was in turn significantly related to lower HRQoL, although with estimates below clinical relevance when adjusted for other symptoms. Prospective studies in men with prostate cancer have identified e.g. pre-treatment sexual distress, neuroticism, and sexual activity [34], as well as therapy modality [31] to be associated with sexual distress at follow-up 1 year after treatment, and pre-treatment sexual distress has been associated with post-radiotherapy HRQoL [4]. To the best of our knowledge, only two longitudinal studies in male populations other than prostate cancer have been published, both examining patients with colorectal cancer. In one, pre-treatment depression and lower confidence were identified as predictors of a combined measure of sexual distress and low sexual satisfaction 2 years later, and having a stoma predicted distress/satisfaction at 5-year follow-up [2]. The other study found that sexual distress at baseline (on average 2.5 years post-diagnosis) was associated with lower HRQoL, depression, and poorer relationship quality at 6-months follow-up [5]. Our results indicate that a potential effect on HRQoL might also be due to co-occurring symptoms, as differences in HRQoL between men with and without sexual distress were no longer clinically relevant, when co-occurring symptoms were included in the model. Contrary to many other studies, we did not find sexual distress to be associated with depression, but rather with clinically relevant pain and insomnia, which were only assessed in one (i.e. pain) or none (i.e. insomnia) of the previous studies. Our findings may be driven by the high level of clinical insomnia identified in our sample (Table 1). Further, age was not associated with sexual distress in our study, potentially due to mutually adjusted analyses. In contrast, Nørskov et al. in unadjusted numbers, found sexual distress to be most frequently reported among patients aged 40–65 years compared to both younger and older persons [13], arguing that fewer impairments in younger people and a protective effect of lifelong sexual history in older individuals may contribute to lower perceived distress compared to individuals in middle-age. Knowledge about the predictors of sexual distress, beyond retrospective reports (e.g. [12]) of patients who have undergone different types of treatment, as well as its potential downstream effects, is limited, and prospective research in heterogenous male cancer populations is needed.

In our study, 6–11% of respondents per item reported distress without also reporting impairment. Most of these respondents were not sexually active (60–82% per item), potentially because of impairment. Conversely, between 2 and 19% per item reported impairment without distress. This illustrates the complex nature of sexual problems, as an interplay of impairments with the psychological and relational context. For some, sexual impairments may not be distressing, e.g. if the individual has little personal interest in sexual activity, for others, distressing sexual impairments may lead to sexual inactivity [8]. This underscores the importance of nuanced inquiry by healthcare professionals, covering both impairment and distress, when investigating sexual problems.

Almost one-third of respondents expressed a current interest in a consultation about sexual problems, and among these, few were receiving help. These findings are similar to a Danish study including people with various cancer diagnoses [11], in which 52% of patients experienced an unmet need for help with sexual problems. We found that when more areas of distress were reported, more participants had a wish for a consultation. Asking patients about sexual distress, not only impairment, may be important to ensure that patients in need can be referred for sexual health care.

Strengths of this study include the use of a questionnaire screening for both sexual impairment and distress in all areas of sexual functioning, and the calculation of percentages reporting combinations of distress and impairment. By including data on other symptoms, we were able to assess the association of sexual distress with other potential side and late effects and adjust our analyses of the association between sexual distress and HRQoL to provide more accurate estimates. To the best of our knowledge, most previous studies have investigated men with cancer primarily after surgical treatment. We invited all patients associated with an oncology department, both those in active treatment and those in follow-up, and thereby included the full spectrum of male cancer patients, as seen in clinical workflow. Access to medical records let us validate self-reported diagnoses and minimize misclassification risk.

Nonetheless, our study has certain limitations, including the cross-sectional design which precludes the possibility to establish causality. The relatively low participation rate in the parent study (40%) and the lower response rate to the SCS-M among older patients as well as those with certain diagnoses may bias our results. The SCS-M has only been preliminarily validated [17], although it has been used in studies of male populations with somatic diseases [18], and it lacks items related to intimacy, which also play a role in sexual life. The SCS-M is, however, to the best of our knowledge, the only questionnaire that assesses frequency of impairment as well as sexual distress across all areas. Further, estimates from post-hoc analyses may be biased due to small sample size and it was not possible to determine if patients were in treatment for primary cancer, relapse or a new cancer. Finally, respondents to the parent study who reported receiving help for sexual problems were more likely to complete the SCS-M, potentially introducing bias. However, they constituted less than 10% of the overall study population, limiting their influence.

## Conclusion

In conclusion, we observed a considerable prevalence of sexual distress as well as of sexual impairment among men with different cancers, with subgroups reporting either impairment without distress or distress without impairment. Sexual distress was associated with experiencing clinically relevant pain and insomnia. Very few respondents reported receiving help for sexual problems, although many expressed an interest in receiving a consultation for sexual problems. A greater focus on sexual health by healthcare professionals, including greater attention to sexual distress in addition to impairment, may be necessary, and this focus should extend to all men with cancer, regardless of diagnosis.

## Supplementary Material

Sexual distress among men with cancer – a cross-sectional study

## Data Availability

Data is available upon request to the corresponding author.
