# Supplementary material for: Sexual distress among men with cancer – a cross-sectional study
Source: Acta Oncol. 2025 Feb 9;64:42525. doi: 10.2340/1651-226X.2025.42525 (PMC11833328; doi:10.2340/1651-226X.2025.42525)
Supplement: Sexual distress among men with cancer – a cross-sectional study [file AO-64-42525-s1.pdf]

Supplementary material for
Sexual distress among men with cancer – a cross-sectional study

Jonas Nahavandipour[1], MSc. Med, Christoffer Johansen[1], MD, PhD, Dr. Med. Sci, Annamaria Giraldi[2,3], MD, PhD, Bolette Skjødt Rafn[1], PT, MSc, PhD, Annika von Heymann[1], MSc Psych, PhD

Table S1:
Characteristics associated with responding to the optional SCS-M screener among study participants of the overall late effect study at the Department of Oncology, Rigshospitalet (n=1072).

	OR (95% CI)	P
**Age (years)**		
19-53	1	
54-65	0.6 (0.39-1.05)	0.07
66-74	0.6 (0.34-1.17)	0.14
75-90	0.5 (0.23-0.87)	**0.02**
**Living alone**		
Yes	0.9 (0.62-1.23)	0.44
**Work status**		
Unemployed	1	
Full time employment	0.7 (0.27-1.98)	0.54
Part time employment	0.8 (0.23-2.60)	0.68
Self employed	0.6 (0.19-1.71)	0.32
Retired	0.4 (0.13-1.01)	0.05
On sick leave	0.6 (0.21-1.85)	0.39
Others	0.4 (0.14-1.28)	0.13
**Level of education**		
Higher education (>4 years)	1	
Higher education (3-4 years)	1.0 (0.70-1.40)	0.95
Higher education (1-2 years)	1.2 (0.71-2.04)	0.49
High school	0.6 (0.30-1.08)	0.08
Primary school	0.8 (0.50-1.26)	0.33
Others	0.8 (0.46-1.38)	0.41
**Primary cancer site**		
Prostate	1	
Lung	0.6 (0.27-1.10)	0.09
Colorectal	0.9 (0.39-2.06)	0.79
Esophageal/stomach	0.5 (0.25-0.97)	0.04
Testes	0.7 (0.35-1.53)	0.40
Brain	0.4 (0.19-0.96)	**0.04**
Bladder	0.6 (0.24-1.61)	0.33
Head and Neck	0.6 (0.30-1.24)	0.17
Multiple	0.5 (0.27-0.97)	**0.04**
Others	0.5 (0.27-1.05)	0.07
**Depression**		
Present	1.2 (0.73-1.82)	0.53
**Receiving help for sexual dysfunction**		
Yes	3.7 (1.77-7.68)	<0.01
**Smoking**		
Never Smoked	1	
Former	1.3 (0.95-1.79)	0.10
Current	0.7 (0.47 -1.14)	0.17
**Alcohol (Units per week)**		
None	1	
0-5	1.0 (0.65-1.38)	0.48
> 5	1.2 (0.77-1.76)	0.79
> 12	1.2 (0.74-1.84)	0.52

Note on response rate in parent study: Among all the 8278 invited patients (men and women), those with malignancies of male genitals (OR: 0.75; 95%-CI: 0.61-0.93) were less likely to respond. Further, there was a minimal difference in respondents and non-respondents' age (OR: 1.00; 95% CI: 1:00-1:01).

Table S2: Prevalence of sexual impairment with and without distress among male cancer patients at the Department of Oncology, Rigshospitalet (n=696)

Cancer type	Area	No impairment, no distress (n (%))	Impairment, but no distress (n (%))	No impairment, but distress (n (%))	Impairment and distress (n (%))
Overall<br><br>(n=696)	Lack of desire	358 (51)*	133 (19)	78 (11)	127 (18)
	Reduced erection firmness	392 (56)	81 (12)	64 (9)	159 (22)
	Increased stimulation need	374 (54)	115 (17)	57 (8)	150 (22)
	Early ejaculation	506 (73)	56 (8)	67 (10)	67 (10)
	Lack of orgasm or ejaculation	517 (74)	34 (5)	66 (10)	79 (11)
	Pain during or after intercourse	614 (88)	16 (2)	44 (6)	22 (3)
Testicular<br><br>(n=183)	Lack of desire	108 (59)	30 (16)	9 (5)	36 (20)
	Reduced erection firmness	129 (71)	8 (4)	9 (5)	37 (20)
	Increased stimulation need	115 (63)	21 (12)	6 (3)	41 (22)
	Early ejaculation	137 (75)	11 (6)	9 (5)	26 (14)
	Lack of orgasm or ejaculation	155 (85)	5 (3)	8 (4)	15 (8)
	Pain during or after intercourse	157 (86)	9 (5)	7 (4)	10 (6)
Head and Neck<br><br>(n=69)	Lack of desire	47 (68)	8 (12)	3 (4)	11 (16)
	Reduced erection firmness	48 (70)	8 (12)	4 (6)	9 (13)
	Increased stimulation need	48 (70)	7 (10)	3 (4)	11 (16)
	Early ejaculation	57 (83)	5 (7)	2 (3)	5 (7)
	Lack of orgasm or ejaculation	58 (84)	4 (6)	4 (6)	3 (4)
	Pain during or after intercourse	46 (90)	1 (1)	0 (0)	0 (0)
Esophageal/Stomach<br>(n=57)	Lack of desire	23 (40)	16 (28)	6 (11)	12 (21)
	Reduced erection firmness	29 (51)	9 (16)	2 (4)	17 (30)
	Increased stimulation need	30 (53)	12 (21)	3 (5)	12 (21)
	Early ejaculation	40 (70)	7 (12)	4 (7)	6 (11)
	Lack of orgasm or ejaculation	45 (79)	3 (5)	4 (7)	5 (9)
	Pain during or after intercourse	54 (95)	0 (0)	3 (5)	0 (0)
Lung<br>(n=51)	Lack of desire	21 (41)	15 (29)	6 (12)	9 (18)
	Reduced erection firmness	24 (47)	10 (20)	6 (12)	11 (22)
	Increased stimulation need	19 (37)	16 (31)	7 (14)	9 (18)

	Early ejaculation	34 (67)	9 (18)	5 (10)	3 (6)
	Lack of orgasm or ejaculation	37 (73)	4 (8)	6 (12)	4 (8)
	Pain during or after intercourse	46 (90)	1 (2)	3 (6)	1 (2)
Prostate (n=51)	Lack of desire	24 (47)	4 (8)	14 (28)	9 (18)
	Reduced erection firmness	26 (51)	1 (2)	13 (26)	11 (22)
	Increased stimulation need	25 (49)	4 (8)	16 (31)	6 (12)
	Early ejaculation	36 (71)	1 (2)	11 (22)	3 (6)
	Lack of orgasm or ejaculation	31 (61)	0 (0)	13 (26)	7 (14)
	Pain during or after intercourse	40 (78)	1 (2)	9 (18)	1 (2)
Brain (n=41)	Lack of desire	21 (51)	9 (22)	4 (10)	7 (17)
	Reduced erection firmness	25 (61)	4 (10)	5 (12)	7 (17)
	Increased stimulation need	21 (51)	7 (17)	4 (10)	9 (22)
	Early ejaculation	32 (78)	4 (10)	3 (7)	2 (5)
	Lack of orgasm or ejaculation	27 (66)	3 (7)	4 (10)	7 (17)
	Pain during or after intercourse	39 (95)	0 (0)	2 (5)	0 (0)
Colorectal (n=41)	Lack of desire	21 (51)	7 (17)	6 (15)	7 (17)
	Reduced erection firmness	19 (46)	6 (15)	5 (12)	11 (27)
	Increased stimulation need	17 (42)	9 (22)	5 (12)	10 (24)
	Early ejaculation	28 (68)	4 (10)	6 (15)	3 (7)
	Lack of orgasm or ejaculation	28 (68)	3 (7)	5 (12)	5 (12)
	Pain during or after intercourse	37 (90)	0 (0)	3 (7)	1 (2)
Other (n=203)	Lack of desire	93 (46)	44 (22)	30 (15)	36 (18)
	Reduced erection firmness	92 (45)	35 (17)	20 (10)	56 (28)
	Increased stimulation need	99 (49)	39 (19)	13 (6)	52 (26)
	Early ejaculation	142 (70)	15 (7)	27 (13)	19 (9)
	Lack of orgasm or ejaculation	136 (67)	12 (6)	22 (11)	33 (16)
	Pain during or after intercourse	173 (85)	4 (2)	17 (8)	9 (4)

* Percentages may not add up to 100 due to rounding

Table S3: Prevalences of sexual impairment, sexual distress and combination hereof in any area among patients in active treatment (N=151) and not in active treatment (N=545) at the department of Oncology, Rigshospitalet.

	**Active treatment** % (N)*	**Not in active treatment** % (N)*	**Overall** % (N)*
**Distress** (on one or more items)	39 (59)	42 (229)	41 (288)
**Impairment** (on one or more items)	60 (91)	60 (329)	60 (420)
**Impairment and distress** (combined on one or more items)	27 (41)	35 (193)	34 (234)

*Numbers are not exclusive and therefore do not add up to the total

Table S4: Odds ratios for sexual distress among men in active treatment with complete data at the Department of Oncology, Rigshospitalet (n=118*)

	OR** (95% CI***)	P
**Age (years)**	1.0 (0.96 – 1.05)	
**Living alone**		
No	Ref	
Yes	0.8 (0.25 – 2.73)	0.75
**Level of education**		
Higher education (>4 years)	Ref	
Higher education (3-4 years)	0.6 (0.20 – 1.66)	0.30
Higher education (1-2 years)	0.1 (0.02 – 0.57)	**0.01**
Primary and high school	0.1 (0.01 – 1.24)	0.08
Others	0.6 (0.03 – 10.45)	0.72
**Primary cancer site**		
Testicular*****	NA	NA
Head and Neck*****	NA	NA
Esophageal/stomach	1.0 (0.15 – 7.14)	0.97
Lung	0.2 (0.03 – 1.67)	0.15
Prostate	Ref	
Brain	0.8 (0.06 – 9.81)	0.60
Colorectal	0.6 (0.08 – 4.79)	0.63
Bladder	1.7 (0.24 – 11.97)	0.62
Multiple	0.7 (0.11 – 4.88)	0.76
Others	0.4 (0.06 – 2.31)	0.28
**Time since first cancer diagnosis (years)**	1.1 (0.85 – 1.36)	0.53
**Pain**		
Present	6.2 (1.71 – 22.54)	**0.01**
**Fatigue**		
Present	0.3 (0.07 – 1.12)	0.07
**Depression**		
Present	0.4 (0.06 – 2.03)	0.25
**Fear of recurrence**		
Present	1.2 (0.37 – 3.62)	0.80
**Insomnia**		
Present	4.7 (1.39 – 15.61)	**0.01**

*Due to outliers, nine participants were removed, **Mutually adjusted, ***Confidence intervals, **** Primary and high school were collapsed due to low sample size, ***** Due to low sample size, categories were collapsed into “Other”.

Table S5: Odds ratios for sexual distress among men not in active treatment with complete data at the Department of Oncology, Rigshospitalet (n=498)

	OR* (95% CI**)	P
**Age (years)**		
	1.0 (0.97 – 1.01)	0.31
**Living alone**		
No	Ref	
Yes	1.0 (0.60 – 1.54)	0.88
**Level of education**		
Higher education (>4 years)	Ref	
Higher education (3-4 years)	1.1 (0.67 – 1.68)	0.80
Higher education (1-2 years)	0.9 (0.43 – 1.71)	0.66
High school	0.8 (0.32 – 1.97)	0.62
Primary school	1.5 (0.73 – 3.21)	0.25
Others	1.5 (0.60 – 3.89)	0.38
**Primary cancer site**		
Testicular	0.7 (0.27 – 2.01)	0.54
Head and Neck	0.5 (0.18 – 1.42)	0.20
Esophageal/stomach	1.3 (0.42 – 3.79)	0.68
Lung	0.8 (0.27 – 2.32)	0.66
Prostate	Ref	
Brain	0.9 (0.31 – 2.88)	0.92
Colorectal	0.9 (0.26 – 2.91)	0.81
Bladder	1.3 (0.37 – 4.86)	0.65
Multiple	1.2 (0.47 – 3.33)	0.66
Others	0.7 (0.24 – 1.86)	0.44
**Time since first cancer diagnosis (years)**	1.0 (0.98 – 1.06)	0.38
**Pain**		
Present	1.8 (1.12 – 2.94	**0.02**
**Fatigue**		
Present	0.8 (0.47 – 1.41)	0.46
**Depression**		
Present	1.7 (0.90 – 3.31)	0.10
**Fear of recurrence**		
Present	1.3 (0.81 – 2.23)	0.26
**Insomnia**		
Present	1.9 (1.24 – 2.82)	**<0.01**

*Mutually adjusted, **Confidence intervals

Figure S1: Distribution of sexual distress and impairment among 696 men with cancer at the Department of Oncology, Rigshospitalet

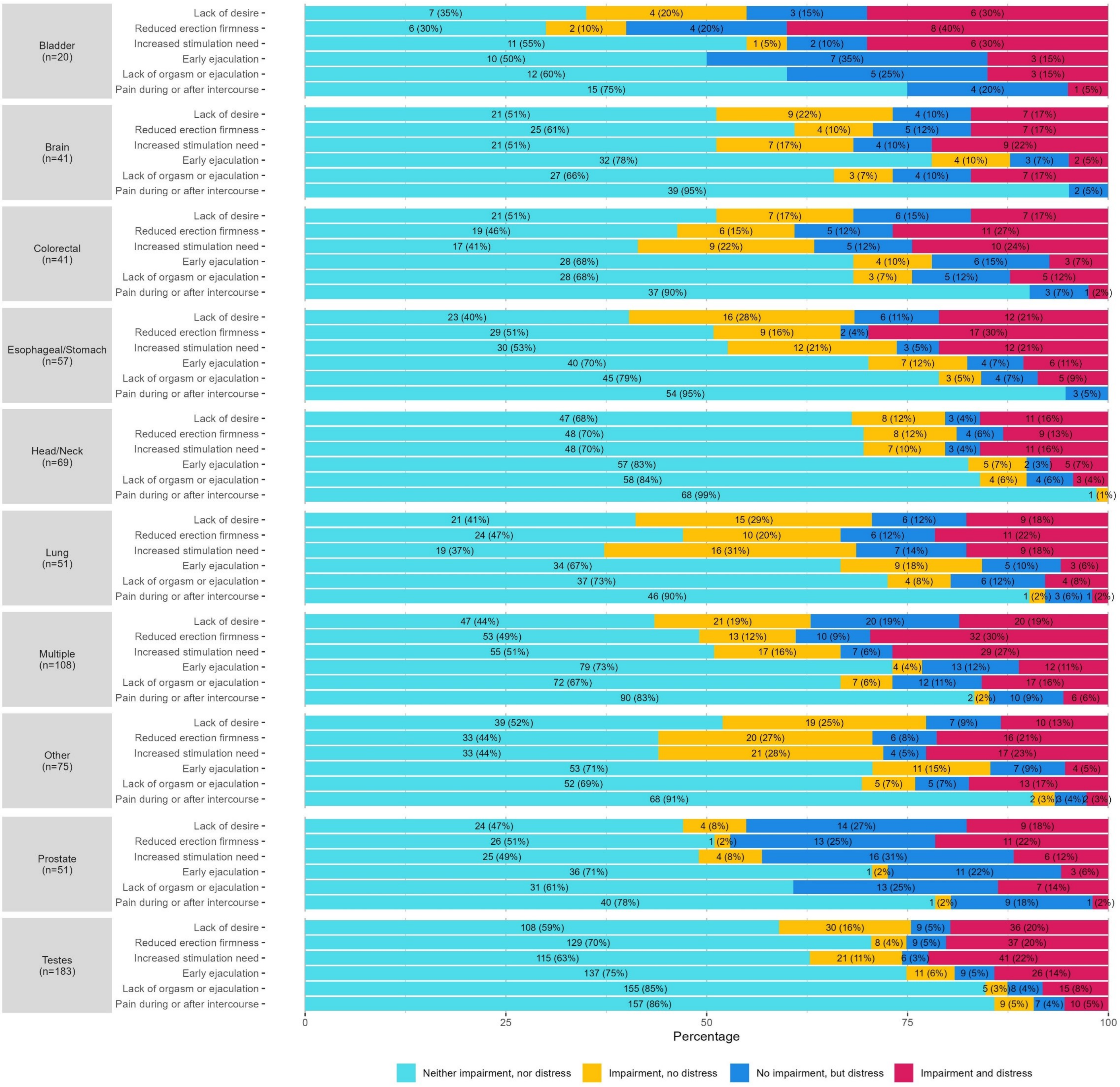

Figure S2:

Title: Distribution of sexual distress and impairment among 151 men in active cancer treatment at the Department of Oncology, Rigshospitalet

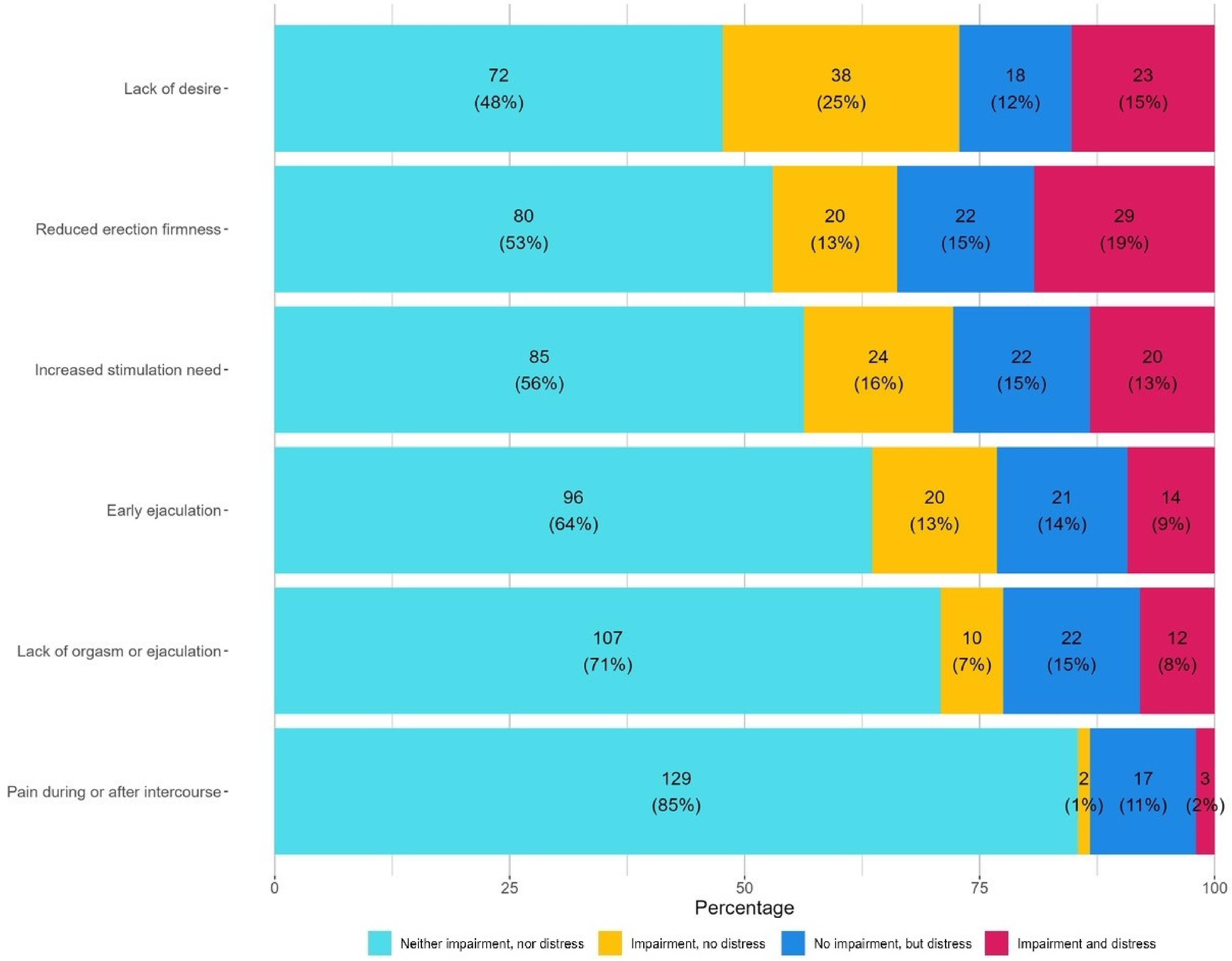

Figure S3:

Title: Distribution of sexual distress and impairment among 545 men not in active treatment for cancer at the Department of Oncology, Rigshospitalet.

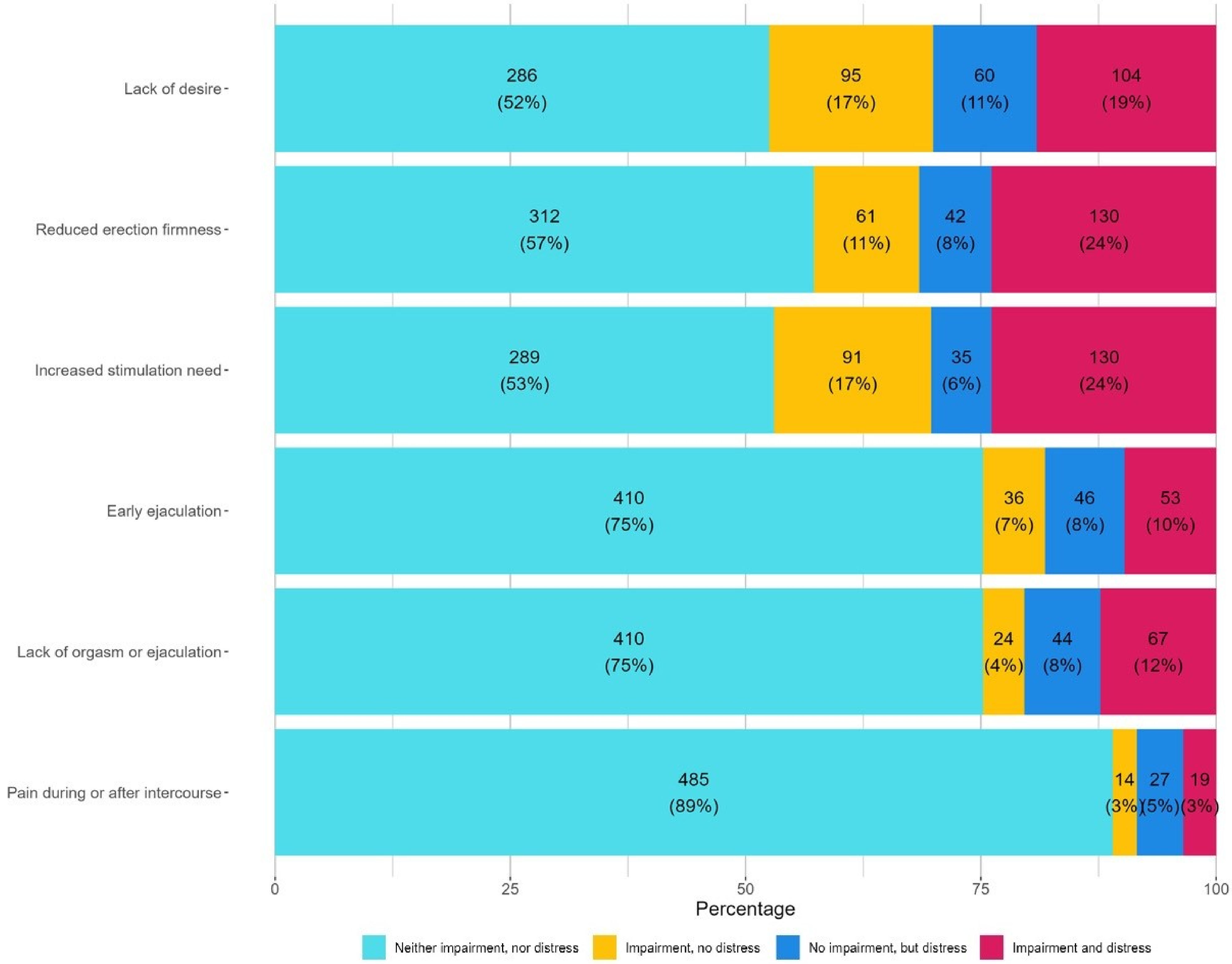

Figure S4: Number of areas with sexual distress and interest in consultations for sexual problems among men with cancer at the Department of Oncology, Rigshospitalet (n=696)

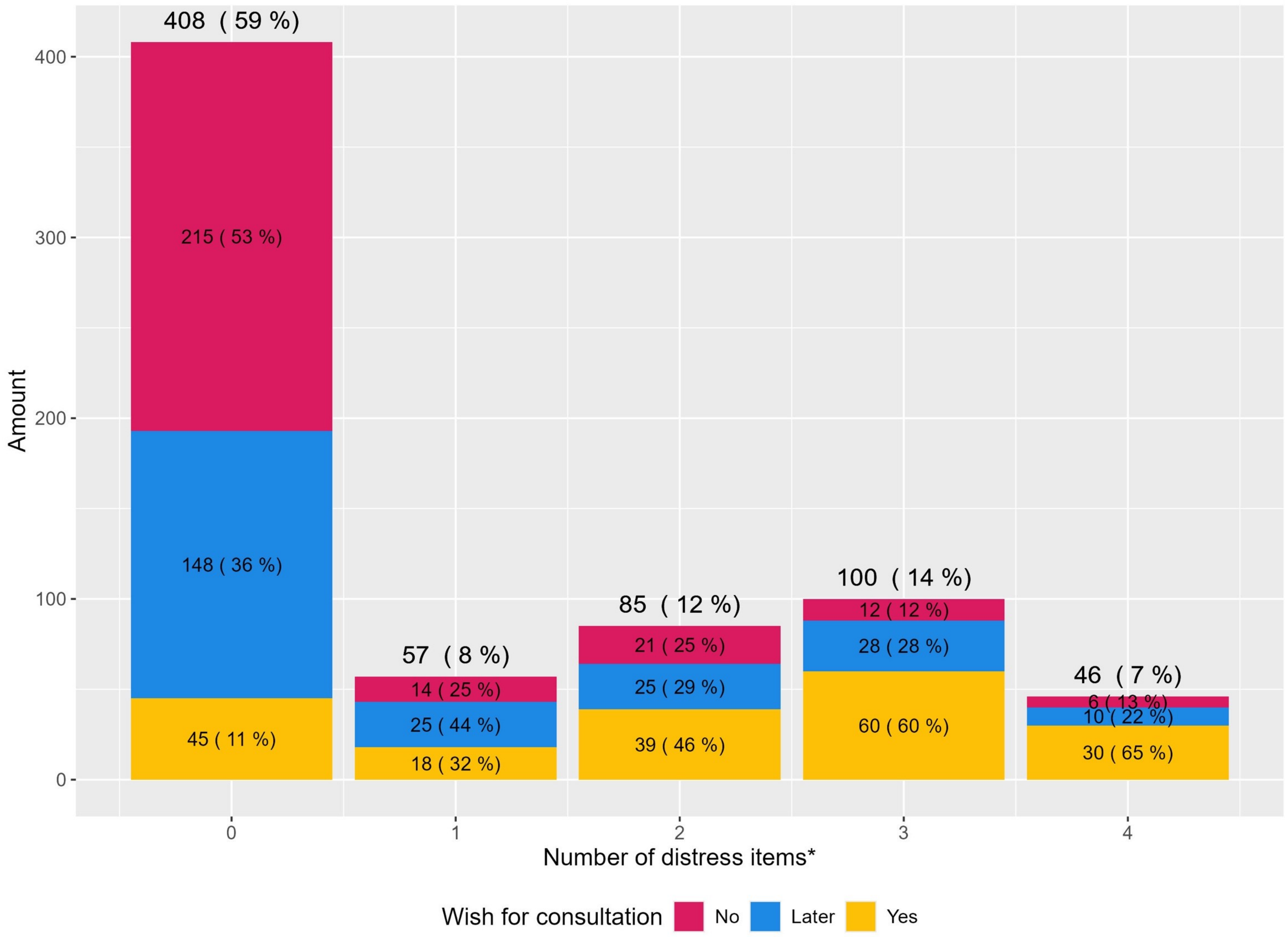

*Questions regarding erection firmness and increased stimulation were considered one items, whilst questions regarding premature ejaculation, lack of orgasm and ejaculation were considered one item

References to questionnaire validation studies and studies previously using the measures employed in Danish populations for questionnaires used in the present study:

Validation of Major Depression Inventory in Danish:

Bech, P., Rasmussen, N.-A., Olsen, L. R., Noerholm, V., & Abildgaard, W. (2001). The sensitivity and specificity of the Major Depression Inventory, using the Present State Examination as the index of diagnostic validity. *Journal of Affective Disorders*, *66*(2–3), 159–164. https://doi.org/10.1016/S0165-0327(00)00309-8

Preliminary validation of the Insomnia Severity Index in Danish:

Dieperink, K. B., Elnegaard, C. M., Winther, B., Lohman, A., Zerlang, I., Möller, S., & Zangger, G. (2020). Preliminary validation of the insomnia severity index in Danish outpatients with a medical condition. *Journal of Patient-Reported Outcomes*, *4*(1), 18. https://doi.org/10.1186/s41687-020-0182-6

Example of use of the Brief Pain Inventory in a study of a sample of Danish patients with cancer:

Grosen, K., Laue Petersen, G., Pfeiffer-Jensen, M., Hoejsgaard, A., & Pilegaard, H. K. (2013). Persistent post-surgical pain following anterior thoracotomy for lung cancer: A cross-sectional study of prevalence, characteristics and interference with functioning†. *European Journal of Cardio-Thoracic Surgery*, *43*(1), 95–103. https://doi.org/10.1093/ejcts/ezs159

Preliminary validation of the Fear of Cancer Recurrence Inventory in Danish:

Hovdenak Jakobsen, I., Jeppesen, M. M., Simard, S., Thaysen, H. V., Laurberg, S., & Juul, T. (2018). Initial validation of the Danish version of the Fear of Cancer Recurrence Inventory (FCRI) in colorectal cancer patients. *Journal of Cancer Survivorship*, *12*(6), 723–732. https://doi.org/10.1007/s11764-018-0709-5

Example of use of the European Organisation for Research and Treatment of Cancer Quality of Life Questionnaire C30 in a study of a sample of Danish patients with cancer:

Rossen, P., Pedersen, A., Zachariae, R., & Maase, H. (2009). Health-Related Quality of Life in Long-Term Survivors of Testicular Cancer. *Journal of Clinical Oncology : Official Journal of the American Society of Clinical Oncology*, *27*, 5993–5999. https://doi.org/10.1200/JCO.2008.19.6931
